# Brazilian Portuguese version of the Amsterdam infant stool scale: a valid and reliable scale for evaluation of stool from children up to 120 days old

**DOI:** 10.1186/s12887-021-02527-0

**Published:** 2021-02-04

**Authors:** Laura Cantisano de Deus Silva, Priscila Monaro Bianchini, Erika Veruska Paiva Ortolan, Juliana Fattori Hamamoto, Rosemary Fermiano, Rebeca Mayara Padilha Rego, João César Lyra, Marc Alexander Benninga, Pedro Luiz Toledo de Arruda Lourenção

**Affiliations:** 1 Botucatu Medical School, São Paulo State University (UNESP), São Paulo, Brazil; 2 Department of Surgery and Orthopedics - Division of Pediatric Surgery, Botucatu Medical School, São Paulo State University (UNESP), São Paulo, Brazil; 3 Department of Pediatrics, Division of Neonatology, Botucatu Medical School, São Paulo State University (UNESP), São Paulo, Brazil; 4 Emma Children’s Hospital, Amsterdam UMC, University of Amsterdam, Pediatric Gastroenterology, Hepatology and Nutrition, Amsterdam, The Netherlands

**Keywords:** Translations, Reproducibility of results, Defecation, Infant, Newborn

## Abstract

**Background:**

For newborns and infants wearing diapers the difficulties in characterizing the appearance of the stool are significant, since the changes in consistency, quantity, and color of the stool are higher than in other age groups. The Amsterdam Infant Stool Scale (AISS) was created and validated in 2009, providing a specific tool for the evaluation of the stool of children up to 120 days old. However, to be used in clinical practice and scientific investigations in Brazil, it is mandatory to perform the translation and cross-cultural adaptation process for Brazilian Portuguese language. Thus, we aim to perform the translation and cross-cultural adaptation of AISS into Brazilian Portuguese and to evaluate the psychometric properties of the translated version.

**Methods:**

The process of translation and cross-cultural adaptation was performed according to the internationally accepted methodology, including: translation, summary of translations, backtranslation, preparation of the pre-final version, application of the pre-test and determination of the final version. The evaluation of the psychometric properties was performed through the application of Brazilian Portuguese AISS, by five examiners (including child health field specialists and a literate adult lay on the subject), analyzing 238 stool photographs of children under 120 days old. The intra and inter-examiner agreement values were determined using kappa statistic. The validity of the criterion was investigated through correlation analysis (Kendall’s coefficient) between the classifications determined by the non-specialist examiner and the expert examiners.

**Results:**

In all 30 tests performed between different examiners, there was an agreement considered as at least moderate (kappa values above 0.40). The intra-examiner reliability was considered as substantial (kappa> 0.6). There was a statistically significant correlation (*p* <  0.05) between the classifications determined by the examiners considered as specialists and the examiner considered as non-specialist.

**Conclusion:**

The Brazilian Portuguese AISS version proved to be valid and reliable to be used by healthcare professionals and the general public in the evaluation of stool from children up to 120 days old.

**Supplementary Information:**

The online version contains supplementary material available at 10.1186/s12887-021-02527-0.

## Background

For newborns and infants wearing diapers the difficulties in characterizing the appearance of the stool are significant, since the changes in consistency, quantity, and color of the stool are higher than in other age groups [1–4]. The gestational age, the degree of maturation of the gastrointestinal tract, the type of diet administered and the presence of possible congenital malformations, such as some hepatic diseases that cause alterations in the color of stool, influence the wide variation of the intestinal habit of children in these age groups [1, 2]. Thus, in 2009, the Amsterdam Infant Stool Scale (AISS) was created and validated, providing a specific tool for the evaluation of the stool of children up to 120 days old [1]. The AISS allows the evaluation of stool consistency, quantity, and color through the interpretation of a series of images of stool in diapers. The amount of stool should be analyzed from the percentage of the occupied diaper, which facilitates and standardizes the analysis [1, 2]. It can be applied for stool evaluation by parents, caregivers, and healthcare professionals. The AISS proved to be more useful to evaluate the bowel pattern of children who still use diapers, compared to the Bristol Stool Form Scale (BSFS) [2] and its use has also been increasing [5–8]. However, to be used in clinical practice and scientific investigations in Brazil, it is mandatory to perform the translation and cross-cultural adaptation process for Brazilian Portuguese language [9–11]. Therefore, we carried out translation and cross-cultural adaptation of AISS into Brazilian Portuguese and evaluated the psychometric properties of the translated version.

## Methods

This was a single-center study, developed at the Botucatu Medical School, São Paulo State University (UNESP), between September 2017 and September 2019. First, the process of translation and cross-cultural adaptation of the AISS to Brazilian Portuguese was performed (Step 1). Subsequently, the evaluation of the psychometric properties of the translated version (Step 2) was performed through application and evaluation by five examiners utilizing 238 stool photographs of children under 120 days old.

The stool photographs were obtained from the stools of children up to 120 days old, including term and premature infants who were in the maternity ward and neonatal unit of a tertiary hospital, and healthy children who were in outpatient care at the Pediatric Outpatient Clinic. This study was approved by the local Research Ethics Committee (protocol number 69504517.9.0000.5411).

### Step 1: translation and cross-cultural adaptation

Aiming to maintain the quality of the cultural adaptation process, the scale was translated and adapted according to the internationally recommended methodology [9–11], incurring six phases:

#### Phase 1: translation

This phase consisted of two translations from the original language (English) into the target language (Brazilian Portuguese). These translations were carried out, independently, by two bilingual translators, whose mother tongue was Brazilian Portuguese.

#### Phase 2: summary of translations

The synthesis meeting was held with the participation of two translators, together with a committee of experts, composed of professionals with experience in the field of children’s health (3 doctors, 1 nurse, 1 psychologist) and a university professor, with experience in cross-cultural adaptation of health assessment instruments.

#### Phase 3: Backtranslation

The synthesized version was translated back into English by two translators who had not participated in the first stage and did not belong to the health field. These translators were mother tongue English speakers and were not informed of the concepts explored by the instrument. These two translations were done independently, without knowledge of the original version of the scale.

#### Phase 4: pre-final version

The pre-final version was built after evaluation and discussion by all translators and the expert committee. The backtranslations were confronted with the original version of the scale. The committee’s function was to analyze the translated versions and develop the pre-final version.

#### Phase 5: application of the pre-test and assessment of the degree of understanding

The pre-test was applied to a sample of 40 adults, 20 healthcare professionals and 20 adults who were literate and did not work in the health field [9–14]. These participants each evaluated a stool photograph of a newborn by applying the translated version of AISS. A five-point Verbal Numerical Scale (VNS) was then applied to assess how easily the translated version of the scale as a whole and each of its three components (quantity, consistency, and color) was understood. The guiding question to evaluate of the translated scale as a whole was: “Did you understand what was asked and the differences between these types of stool?”, and to evaluate each of the components was: “Did you understand the differences between these types of stool according to this component of the scale?” The minimum ascribed value was zero (“I did not understand anything”) and the maximum value was five (“I understood perfectly and have no doubts”). Values below three were considered to indicate insufficient understanding [11–13]. These data were tabulated and the median values (minimum/maximum) were calculated. The questions with more than 15% of values considered of insufficient comprehension would have to be reformulated by the expert committee and applied to new respondents [11, 14] Potential differences between the two groups of participants in this phase were also analyzed.

#### Phase 6: evaluation of results and obtaining the final version

This phase consisted of the analysis of the results obtained in the pre-test, by the members of the expert committee. From the discussion of the items that still had some difficulty of understanding by the population evaluated, with minimal modifications, the final version of Brazilian Portuguese AISS (BP-AISS) was created.

### Step 2: psychometric properties assessment

A total of 238 photographic images were taken of stools from children up to 120 days old, who had no metabolic disorders, congenital malformations, or gastrointestinal disorders and who had not undergone gastrointestinal surgery. The photographs were taken during the daytime period, by three researchers, with the same digital camera (zoom lens, original magnification × 4 and × 7.2 megapixels) [1]. The diapers with the stool were positioned at 20 cm from the digital camera. The camera’s macro function was applied to all photos. To photograph fresh stool, nurses informed researchers every four hours about the bowel movements of all children in the hospital or an outpatient clinic.

The evaluation of the psychometric properties of the BP-AISS included tests to assess the reliability and validity of criteria. For this, BP-AISS was applied for evaluation of the 238 photographs obtained by five examiners: Examiner 1 was a pediatric surgeon; Examiner 2 was a neonatologist; Examiner 3 was a literate adult woman with completed higher education but without professional experience of child healthcare; Examiner 4 was a nurse working in the neonatal unit, and Examiner 5 was a last year graduate medical student. Examiners who were specialists in children’s health (Examiners 1, 2, and 4) had at least 10 years of professional experience.

The reliability of the translated scale was investigated by comparing the results of the evaluations of the photographs performed by each of the five examiners (inter-examiners reliability), and by the agreement between the evaluations performed by Examiner 5, at two different moments after 3 months (intra-examiner reliability), to investigate the reproducibility of the scale. The validity of the criterion was investigated through correlation analysis between the classifications determined by the non-specialist examiner (Examiner 3) and by the expert examiners, with professional performance in the child health field (Examiners 1, 2, and 4), whose evaluations were considered the “gold standard”.

### Statistical analysis

The sample size for the evaluation of psychometric properties of the BP-AISS was calculated from the highest value of agreement between examiners (78%), reported in the study of Bekkali et al. (2009) [1], considering a zero value of kappa of 0.50, with test power estimated at 90%, to detect differences of up to 70% for the zero value of kappa.

The agreement values were determined using the kappa statistic, using the kappa estimator with quadratic weights (Fleiss-Cohen), considering the predominantly ordinal character of the scale [15]. The correlation analysis between the responses obtained by the different examiners was performed by Kendall’s correlation coefficient.

Continuous numerical data were expressed as median (minimum/maximum). Continuous numerical variables of non-parametric distribution were evaluated by the Mann-Whitney and Kruskall-Wallis tests, followed by the Dunn post-test. The comparison between the responses in the evaluation of a stool photograph, was performed by the Kolmogorov-Smirnov test. The significance level was 5% and the analysis was performed in the SPSS 22.0 for Windows.

## Results

### Step 1: translation and cross-cultural adaptation

The six phases were completed according to the proposed methodology [9–12]. All versions produced are available [see Additional file 1].

The pre-final version of BP-AISS was applied to a group of 20 healthcare professionals and 20 literate adults unrelated to the health field (lay audience) [see Additional file 2]. The maximum value of participants who declared insufficient comprehension was 5%, below the limit value of 15%. The median of the values of comprehension, obtained by the VNS, was higher than 3.00. There were no statistically significant differences between the two groups of participants for the values determined by the VNS regarding the degree of comprehension of the pre-final version of the translated scale as a whole and for each of its components.

Participants were also asked to evaluate a stool photograph from a 30-day-old child [see Additional file 3], chosen at random, and classify it according to the pre-final version of BP-AISS. In the general classification of BP-AISS, the most used classifications were 3-B-IV, determined by 16 participants (40%), and 4-A-IV, by seven participants (17.5%). There was a more used classification for each of the components of the scale, with values ranging from 62.5% (for type B, in the consistency variable) to 80% (for type IV, in the color variable). Analyzing the variation of a score above or below that determined by the most used classification, we found 100% of the classifications determined for the components of quantity and consistency and 87.5% of the classifications determined for the color component. There were no statistically significant differences in the distribution of the classifications determined by the two groups of participants, for each of the three components of AISS [see Additional file 2].

**Fig. 1:**
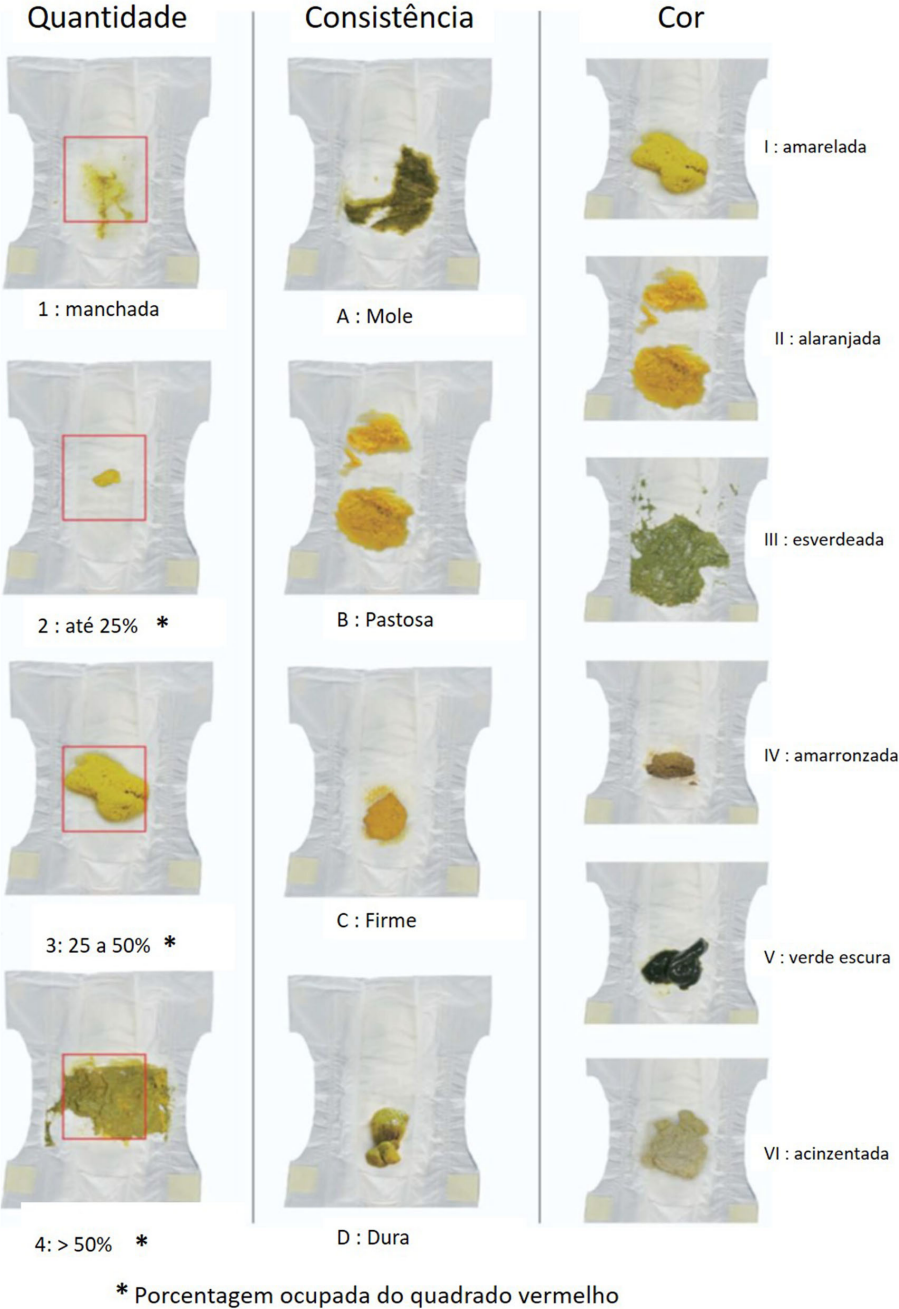
Final version of the BP-AISS

These results were discussed at a new meeting of the expert committee when the few items that still presented some difficulty in understanding by the population evaluated were reviewed. After minimal modifications, the Final Version of BP-AISS was created (Fig. 1).

### Step 2: psychometric properties assessment

The 238 stool photographs were obtained from patients with a median age of 19 days old, with a minimum of 0 and a maximum of 120 days, including term or premature infants. One hundred and nine patients (45.8%) were male, and 129 (54.2%) were female. Seventy- two patients (30.3%) were healthy term newborns in full rooming-in, 71 (29.8%) were healthy preterm newborns gaining weight, 59 (24.8%) were healthy newborns and infants in outpatient clinic routine follow up, and 36 (15.1%) were preterm newborns with respiratory problems.

In the evaluation of the inter-examiners reliability it was observed that, in most of the examiner combinations, more than 50% of the 238 photographs received the same classification by the BP-AISS (Table 1). Also, the proportions of photographs in which the classification established by two examiners varied more than two BP-AISS categories were quite limited, ranging from 0% to a maximum of 10.0%.

**Table 1:** Results of tests performed to evaluate inter-examiners reliability, according to each BP-AISS component

Combinations of examiners	Quantity		Consistency		Color	
	% of photos with the same BP-AISS classifications	% of photos with a variation greater than 2 BP-AISS classifications	% of photos with the same BP-AISS classifications	% of photos with a variation greater than 2 BP-AISS classifications	% of photos with the same BP-AISS classification	% of photos with a variation greater than 2 BP-AISS classifications
**E1 x E2**	64.3	0.8	44.9	0	58.0	7.1
**E1 x E3**	65.5	0.4	45.8	0	56.7	4.2
**E1 x E4**	49.6	1.7	53.4	0.4	50.4	7.1
**E1 x E5**	57.1	0.8	46.2	0.8	55.8	10.0
**E2 x E3**	64.2	0.8	30.3	0	59.7	0.8
**E2 x E4**	53.8	0.4	49.2	0.8	52.9	6.7
**E2 x E5**	66.8	0	42.4	1.3	72.3	5.5
**E3 x E4**	43.7	2.9	40.8	0.4	55.9	5.9
**E3 x E5**	55.4	0.4	58.8	0.8	60.5	1.7
**E4 x E5**	51.6	0.8	52.1	1.3	48.3	9.7

*E1* Examiner 1; *E2* Examiner 2; *E3* Examiner 3; *E4* Examiner 4; *E5* Examiner 5

Table 2 shows the agreement, estimated by the kappa coefficient with quadratic weights, between the BP-AISS classifications established by the different examiners for the stool photographs. In all 30 tests performed (10 tests for each of the 3 components of the AISS), there was an agreement with a magnitude considered at least moderate (kappa values above 0.40), according to the classification proposed by Landis and Koch (1977) [16]. Agreement with magnitude considered as moderate or substantial were obtained both in tests amongst expert examiners (E1, E2, and E4) and in tests amongst expert examiners and the non-expert examiner (E3). Comparing the kappa values obtained, according to each of the three AISS components, it can be observed that the kappa values obtained in the “Consistency” stool evaluation tests were significantly lower (*p* = 0.001; Kruskal-Wallis test) than the values obtained in the “Quantity” (*p* <  0.05; Dunn post-test) and “Color” (p <  0.05; Dunn post-test) stool evaluation tests.

**Table 2:** Inter-examiner reliability: agreement among examiners in the classification of stool photographs, according BP-AISS

Agreement between examiners	Quantity		Consistency		Color	
	k	CI (95%)	k	CI (95%)	k	CI (95%)
**E1 x E2**	0.636	0.475–0.797	0.495	0.372–0.618	0.575	0.396–0.755
**E1 x E3**	0.585	0.413–0.757	0.502	0.369–0.634	0.580	0.403–0.757
**E1 x E4**	0.515	0.391–0.639	0.508	0.347–0.670	0.543	0.376–0.711
**E1 x E5**	0.561	0.398–0.723	0.416	0.284–0.549	0.500	0.330–0.668
**E2 x E3**	0.686	0.553–0.820	0.447	0.357–0.538	0.805	0.755–0.855
**E2 x E4**	0.628	0.519–0.738	0.441	0.317–0.571	0.632	0.486–0.778
**E2 x E5**	0.758	0.656–0.859	0.509	0.396–0.623	0.743	0.592–0.895
**E3 x E4**	0.456	0.350–0.566	0.417	0.337–0.604	0.634	0.480–0.788
**E3 x E5**	0.592	0.448–0.736	0.585	0.149–0.750	0.774	0.677–0.870
**E4 x E5**	0.611	0.498–0.725	0.412	0.268–0.555	0.553	0.404–0.703

k = agreement value, established by the kappa coefficient with quadratic weights

CI (95%) = 95% confidence interval

*E1* Examiner 1; *E2* Examiner 2; *E3* Examiner 3; *E4* Examiner 4; *E5* Examiner 5

There was a statistically significant correlation between the BP-AISS stool photograph classifications determined by the examiners considered as specialists and the examiner considered as non-specialist, as presented in Table 3.

**Table 3:** Correlation between stool classifications according BP-AISS determined by expert examiners and non-expert examiner

	Quantity		Consistency		Color	
	τ (tau)	p *	τ (tau)	p *	τ(tau)	p *
**Examiner 1 x Examiner 3**	0.792	< 0.001	0.757	< 0.001	0.668	< 0.001
**Examiner 2 x Examiner 3**	0.851	< 0.001	0.769	< 0.001	0.857	< 0.001
**Examiner 4 x Examiner 3**	0.794	< 0.001	0.771	< 0.001	0.689	< 0.001

Expert examiners (Examiners 1, 2, and 4); non-expert examiner (Examiner 3)

τ (tau) = Kendall correlation coefficient; * *p* value associated to the Kendall correlation coefficient

The intra-examiner reliability of the BP-AISS was tested by investigating the agreement for the analysis of photographs by the same examiner, after 3 months between evaluations. Examiner 5 was the one who performed these evaluations, obtaining indicators of agreement considered as substantial [16] (kappa> 0.6): quantity: k = 0.634 (0.454–0.782); consistency: k = 0.636 (0.474–0.799); color: k = 0.816 (0.716–0.915).

## Discussion

This was the first time that AISS went through the process of translation and cross-cultural adaptation to a language other than English. The values obtained during the pre-test phase for investigating the degree of understanding were considered satisfactory [9–11]. The pre-final version also proved to be applicable for healthcare professionals and lay adults, with no significant differences between the classifications determined by these two groups of participants.

The evaluation of the psychometric properties of the BP-AISS showed agreement indicators considered satisfactory among the different combinations of examiners [16]. Moreover, we observed a high percentage of identical responses, determined by different examiners, for the same stool photograph evaluated by the translated scale. The percentage of responses that varied more than two classifications on the scale was limited, demonstrating that the same images, when evaluated by the scale, by different individuals, provide close responses. The BP-AISS also proved reproducible, with a substantial agreement, in the analysis of stool photographs by the same examiner at different times. Thus, the tests developed for the investigation of reliability proved that the BP-AISS is reliable, by providing similar results for the same respondent at different times, characterizing stability, and for different examiners, characterizing equivalence, composing the two axes of external reliability [17, 18].

The validity of a criterion represents the relationship between scores for a given instrument and some widely accepted measure, i.e. an instrument or criterion considered to be the “gold standard” [17]. For the evaluation of this psychometric measure, we consider as the “gold standard” measure the expert examiners’ classifications of the stool photographs according to the BP-AISS. We observed that there was a statistically significant correlation between the classifications of the expert examiners and the non-specialist examiner, for the three components of the BP-AISS, suggesting that the scale can provide a measure considered adequate since its results agree with the results of the “gold standard” evaluations.

In the evaluation of the indicators of agreement obtained in the tests performed between different examiners, the “Consistency” component obtained the lowest values, with a statistically significant difference for the “Quantity” and “Color” components. This result is like that described in the original validation study of the scale, in which the “Consistency” component was the one that also presented the lowest rates of agreement [1]. Since the evaluation of consistency is a fundamentally important parameter in evaluating the stool’s aspect, being directly related to the colonic transit time, this can be considered as a limitation of AISS. Possibly, this limitation is related to the evaluation of stool present in diapers that make it difficult to determine the consistency, especially when compared to the stool present in toilets—the scenario that is commonly measured by BSFS. Especially in stool with a softer consistency, contact with the buttocks, the dispersion over the surface of the diaper, and the time interval between both the bowel movement and the evaluation are factors that can substantially alter the evaluation of consistency [3]. This potential limitation can be minimized by performing a direct evaluation of the stool in the diapers without the use of photographs. Wojtyniak et al. (2018) [8] obtained indicators of agreement among examiners with higher values, for the three components of AISS, when the analysis was performed directly in the diapers and not through the evaluation of photographs. These limitations related to the evaluation of the consistency of stool by AISS have been one of the arguments used by authors who propose new graphic scales for the evaluation of stool in children in this age group. Recently, Huysentruyt et al. (2019) [3] described a new scale, called the Brussels Scale, which proposes the use of seven types of stool for the determination of consistency, like that proposed by the BSFS. Although the authors found high indicators of agreement between different examiners when comparing the images of the seven types of stool on this scale with the images of the seven types of stool on the BSFS, we believe that AISS allows a more global assessment of the appearance of the stool and the pattern of bowel movement, so peculiar in children of these age groups. In addition to the evaluation of consistency, AISS allows the evaluation of the amount of stool, of relevance in clinical follow-up, for example, in patients who are recovering from intestinal transit after surgical approaches or in treatment for allergic enterocolitis and other gastrointestinal pathologies. The AISS also allows for the evaluation of the stool color, information that is very relevant in the clinical assessment of children of these age groups. For example, for the identification of acholic and hypocholic stool related to obstructive jaundice. In this sense, even healthcare professionals may present difficulties in the identification of acholic or hypocholic stool [19] which reinforces the indication of the clinical use of graphic scales for systematic evaluation of the stool of newborns and infants, to diagnose potential alterations early [20].

Two main limitations of this study should be considered. First, the study was conducted in a single center, which limits generalizations and may bring biases related to the social, economic, and cultural context of the sample. Second, stools were analyzed in photographic images and not directly in the diapers, which can influence the interpretation of the stool’s consistency [8]. However, the analysis of stool photographs is commonly used in validation studies of visual stool form scales since it allows evaluations by different examiners at different times [1, 21–24]. Furthermore, this limitation was minimized by obtaining the photographic images of fresh stools always taken in less than four hours after the bowel movements, according to the methodology used in the AISS development study. Conversely, some strengths of the study can be highlighted, such as the significant number of photographs of diapers analyzed, the evaluation carried out by five different examiners, including healthcare professionals and the lay public.

## Conclusion

For all these reasons, the BP-AISS has proved to be valid and reliable to be used by healthcare professionals and the general public in the evaluation of stool from children up to 120 days old and can be used in clinical practice and scientific investigations.

## Supplementary Information

**Additional file 1.** Versions produced during the Translation and cross-cultural adaptation (Step 1). All versions produced during the Translation and cross-cultural adaptation (Step 1) are available in this file.

**Additional file 2.** Pre-test results. Data from the comparative analysis between the two groups of participants of the Pre-test, according to each component of the scale, to evaluate the degree of understanding and the results of the analysis of a stool photograph by applying the pre-final version of BP-AISS.

**Additional file 3.** Stool photograph used in the pre-test application. Stool photograph from a 30-day-old child used in the pre-test application and assessment of the degree of understanding (Step 1 - Phase 5).

## Data Availability

The datasets used and/or analysed during the current study are available from the corresponding author on reasonable request.

## Abbreviations

AISS: Amsterdam Infant Stool Scale
BSFS: Bristol Stool Form Scale
VNS: Verbal Numerical Scale
BP-AISS: Brazilian Portuguese Amsterdam Infant Stool Scale
E: Examiner
k: agreement value, established by the kappa coefficient with quadratic weights
CI (95%): 95% confidence interval
τ (tau): Kendall correlation coefficient
p: *p* value
