# Supplementary material for: Brazilian Portuguese version of the Amsterdam infant stool scale: a valid and reliable scale for evaluation of stool from children up to 120 days old
Source: BMC Pediatr. 2021 Feb 4;21:64. doi: 10.1186/s12887-021-02527-0 (PMC7860020; doi:10.1186/s12887-021-02527-0)
Supplement: Supplementary file 1 — Additional file 1. Versions produced during the Translation and cross-cultural adaptation (Step 1). All versions produced during the Translation and cross-cultural adaptation (Step 1) are available in this file. [file 12887_2021_2527_MOESM1_ESM.docx]

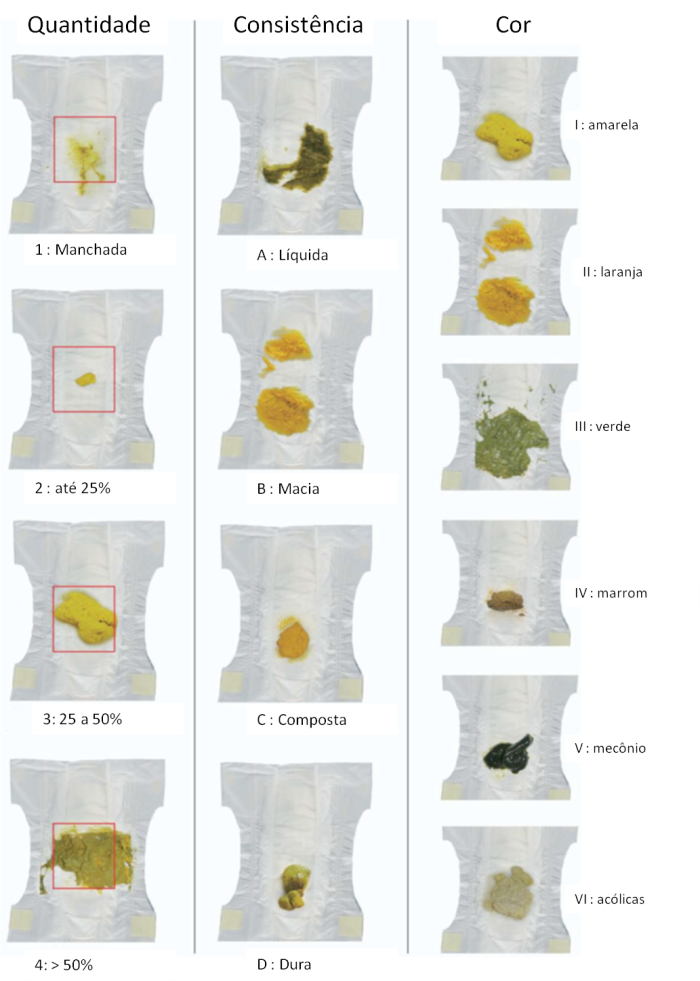


**Phase 1: Translation into Brazilian Portuguese (translation number 1).**


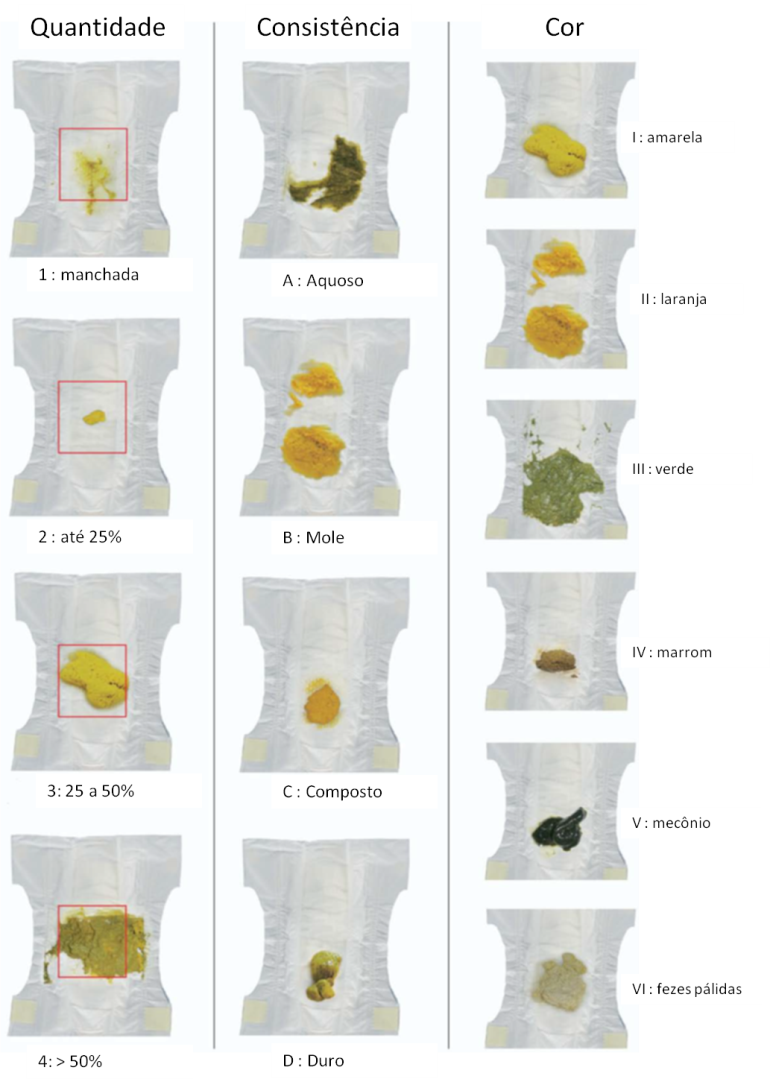


**Phase 1: Translation into Brazilian Portuguese (translation number 2).**


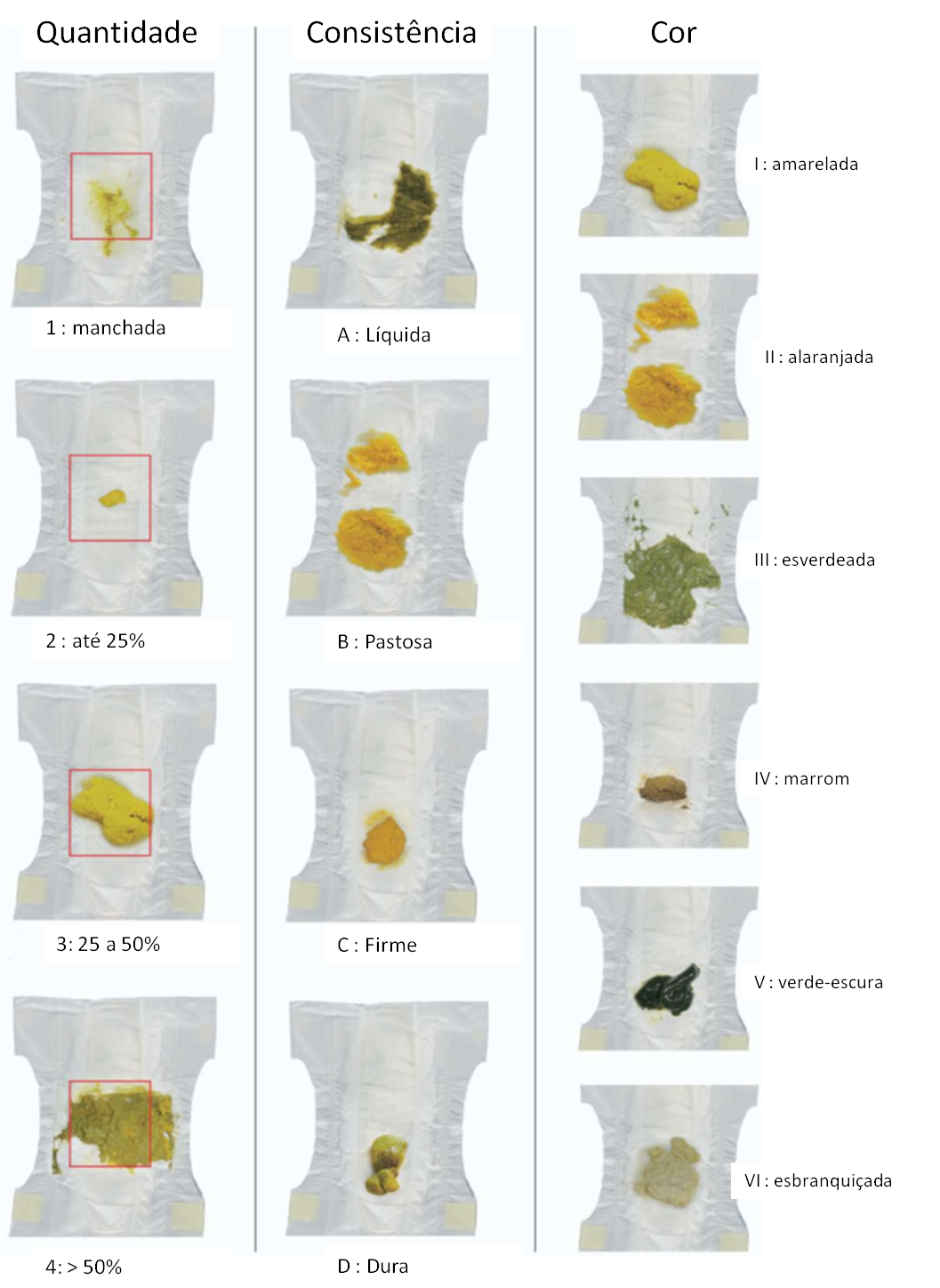


**Phase 2: Summary of translations**


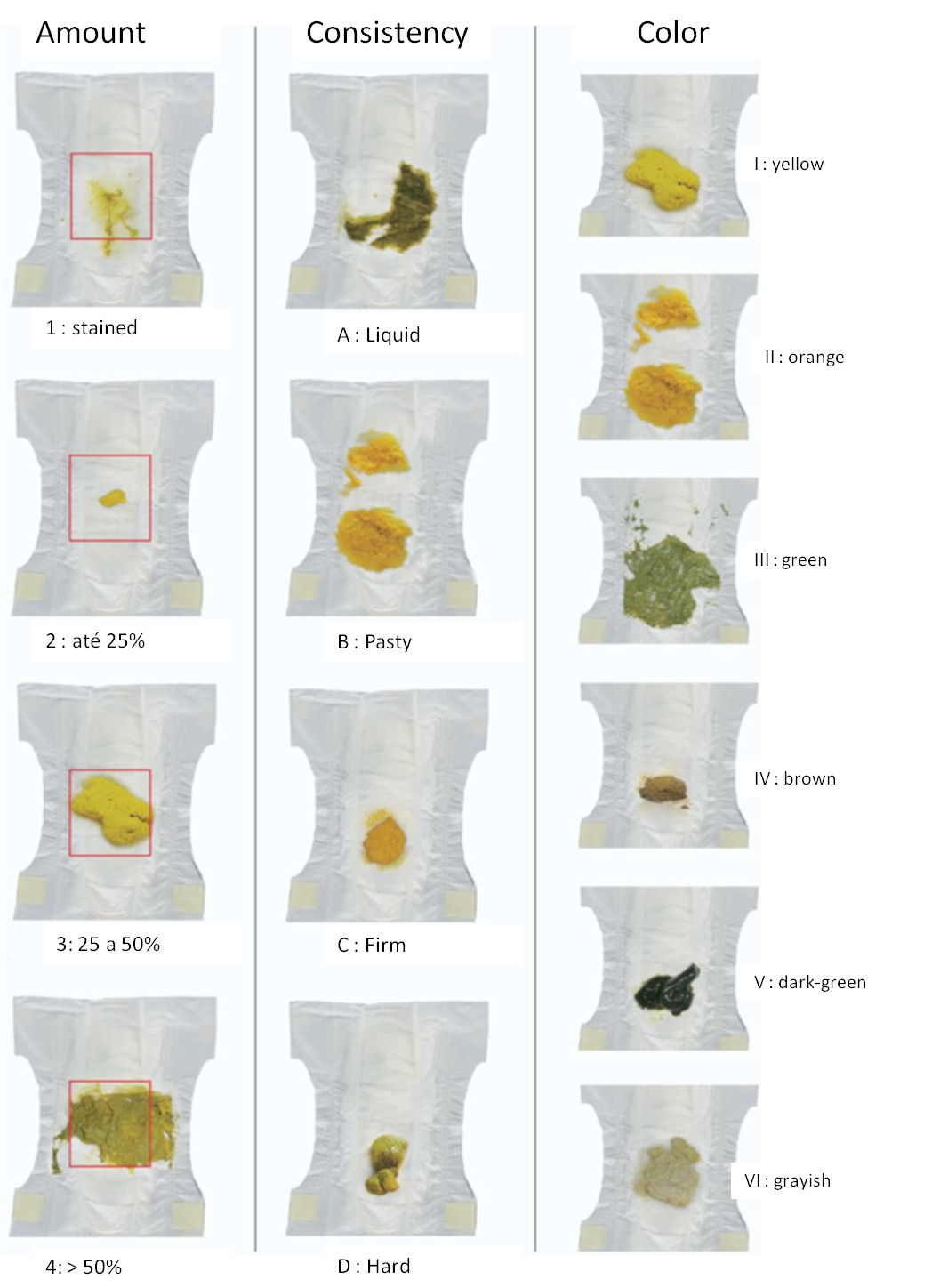


**Phase 3: Backtranslation ( number 1).**


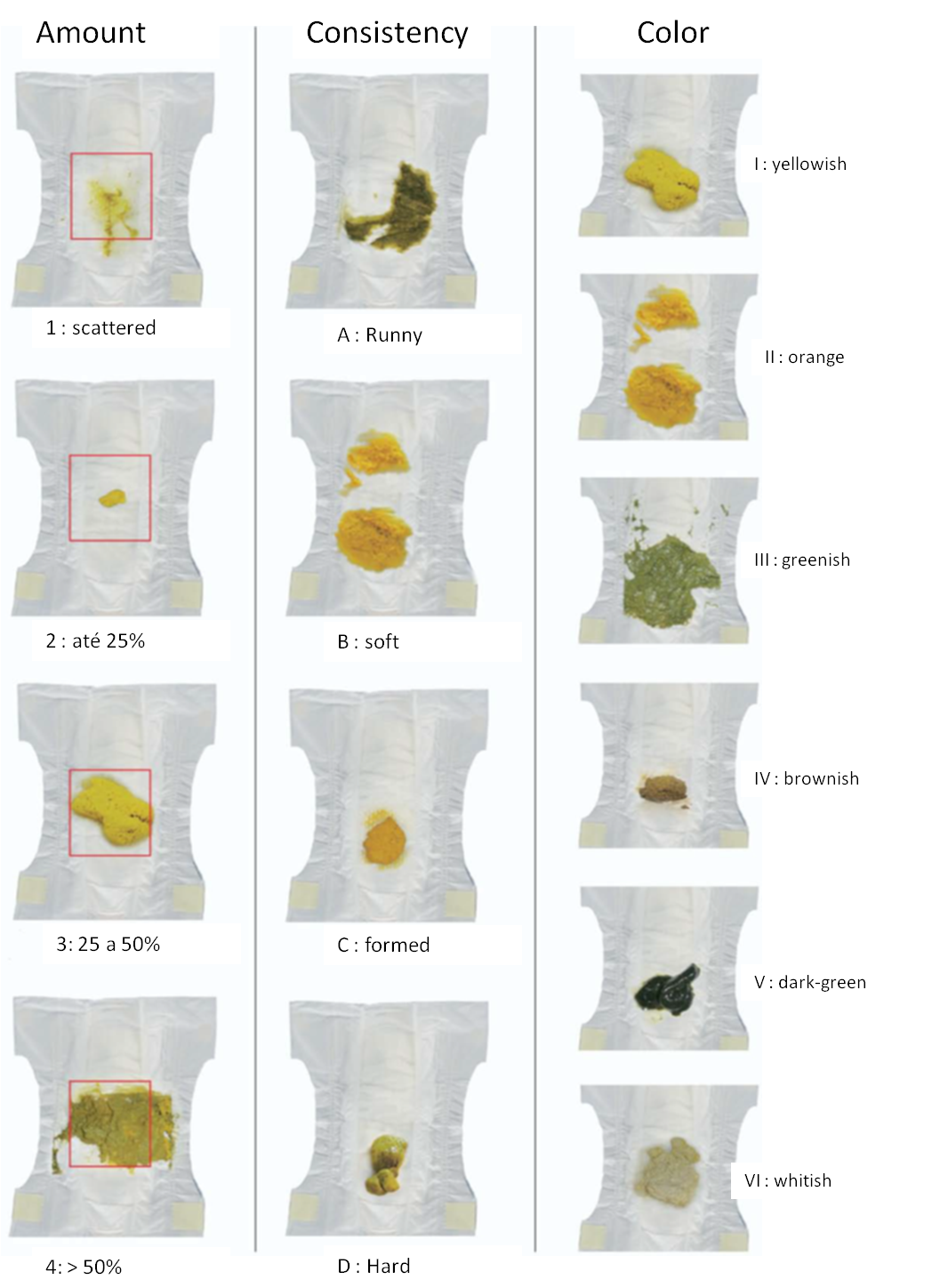


**Phase 3: Backtranslation ( number 2).**


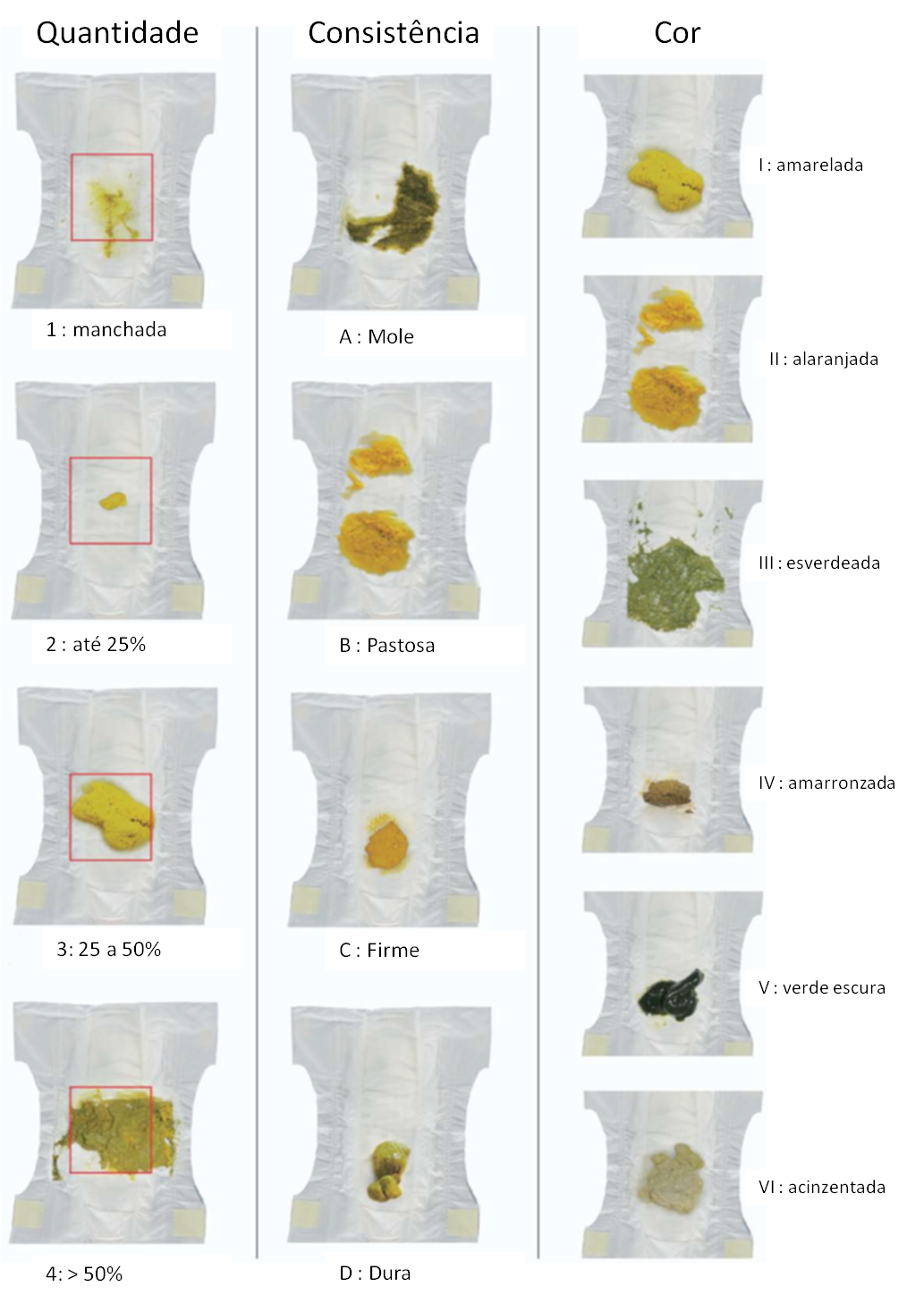


**Phase 4: Pre-final version of the translated scale**
