# Supplementary material for: Brazilian Portuguese version of the Amsterdam infant stool scale: a valid and reliable scale for evaluation of stool from children up to 120 days old
Source: BMC Pediatr. 2021 Feb 4;21:64. doi: 10.1186/s12887-021-02527-0 (PMC7860020; doi:10.1186/s12887-021-02527-0)
Supplement: Supplementary file 2 — Additional file 2. Pre-test results. Data from the comparative analysis between the two groups of participants of the Pre-test, according to each component of the scale, to evaluate the degree of understanding and the results of the analysis of a stool photograph by applying the pre-final version of BP-AISS. [file 12887_2021_2527_MOESM2_ESM.docx]

**Pre-test results: a comparative analysis between the two groups of participants, according to each component of the scale, to evaluate the degree of understanding and the results of the analysis of a stool photograph by applying the pre-final version of BP-AISS.**

|  | | **Healthcare professionals (n=20)** | | **Lay audience (n=20)** | |  |
| --- | --- | --- | --- | --- | --- | --- |
| **Evaluation of the degree of understanding of the pre-final version** | | **% of individuals with insufficient understanding** | **VNS Median values (min-max)** | **% of individuals with insufficient understanding** | **VNS Median values (min-max)** | **p *** |
| **Pre-final version** | | 0 | 5 (4-5) | 0 | 5 (3-5) | 0,704 |
| **Component - quantity** | | 5% | 5 (2-5) | 0 | 5 (3-5) | 0,913 |
| **Component - consistency** | | 5% | 5 (2-5) | 5% | 5 (2-5) | 0,533 |
| **Component - color** | | 0 | 5 (3-5) | 0 | 5 (3-5) | 0,465 |
| **Evaluation of the stool photograph according to the pre-final version** | | **Number of responses** | **% of responses per AISS component** | **Number of responses** | **% of responses per AISS component** | **p^#^** |
| **Component - quantity** | **1** | 0 | 0 | 0 | 0 | >0,05 |
|  | **2** | 1 | 5% | 1 | 5% |  |
|  | **3** | 14 | 70% | 14 | 70% |  |
|  | **4** | 5 | 25% | 5 | 25% |  |
| **Component - consistency** | **A** | 8 | 40% | 7 | 35% | >0,05 |
|  | **B** | 12 | 60% | 13 | 65% |  |
|  | **C** | 0 | 0 | 0 | 0 |  |
|  | **D** | 0 | 0 | 0 | 0 |  |
| **Component - color** | **I** | 0 | 0 | 2 | 10% | >0,05 |
|  | **II** | 0 | 0 | 3 | 15% |  |
|  | **III** | 1 | 5% | 0 | 0 |  |
|  | **IV** | 19 | 95% | 13 | 65% |  |
|  | **V** | 0 | 0 | 2 | 10% |  |
|  | **VI** | 0 | 0 | 0 | 0 |  |

VNS: Verbal Numerical Scale

* p value relative to the Mann-Whitney test.

^#^ p value relative to the Kolmogorov-Smirnov test.
