# Supplementary figures and images for: Brazilian Portuguese version of the Amsterdam infant stool scale: a valid and reliable scale for evaluation of stool from children up to 120 days old
Source: BMC Pediatr. 2021 Feb 4;21:64. doi: 10.1186/s12887-021-02527-0 (PMC7860020; doi:10.1186/s12887-021-02527-0)

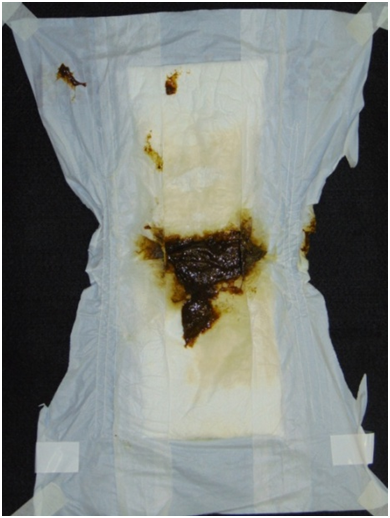

Supplement: Supplementary file 3 — Additional file 3. Stool photograph used in the pre-test application. Stool photograph from a 30-day-old child used in the pre-test application and assessment of the degree of understanding (Step 1 - Phase 5). [file 12887_2021_2527_MOESM3_ESM.tif]
